# The inhibitory effect of silencing *CDCA3* on migration and proliferation in bladder urothelial carcinoma

**DOI:** 10.1186/s12935-021-01969-x

**Published:** 2021-05-12

**Authors:** Dexin Shen, Yayun Fang, Fenfang Zhou, Zhao Deng, Kaiyu Qian, Gang Wang, Yu Xiao, Lingao Ju, Xinghuan Wang

**Affiliations:** 1 Department of Urology, Zhongnan Hospital of Wuhan University, Wuhan, China; 2 Department of Biological Repositories, Zhongnan Hospital of Wuhan University, Wuhan, China; 3 Human Genetic Resource Preservation Center of Hubei Province, Wuhan, China; 4 Wuhan Research Center for Infectious Diseases and Cancer, Chinese Academy of Medical Sciences, Wuhan, China; 5 Medical Research Institute, Wuhan University, Wuhan, China

**Keywords:** CDCA3, Bladder urothelial carcinoma, Migration, cell cycle, p21

## Abstract

**Background:**

CDCA3 is an important component of the E3 ligase complex with SKP1 and CUL1, which could regulate the progress of cell mitosis. *CDCA3* has been widely identified as a proto-oncogene in multiple human cancers, however, its role in promoting human bladder urothelial carcinoma has not been fully elucidated.

**Methods:**

Bioinformatic methods were used to analyze the expression level of *CDCA3* in human bladder urothelial carcinoma tissues and the relationship between its expression level and key clinical characteristics. In vitro studies were performed to validate the specific functions of CDCA3 in regulating cell proliferation, cell migration and cell cycle process. Alterations of related proteins was investigated by western blot assays. In vivo studies were constructed to validate whether silencing *CDCA3* could inhibit the proliferation rate in mice model.

**Results:**

Bioinformatic analysis revealed that *CDCA3* was significantly up-regulated in bladder urothelial carcinoma samples and was related to key clinical characteristics, such as tumor grade and metastasis. Moreover, patients who had higher expression level of *CDCA3* tend to show a shorter life span. In vitro studies revealed that silencing *CDCA3* could impair the migration ability of tumor cells via down-regulating EMT-related proteins such as MMP9 and Vimentin and inhibit tumor cell growth via arresting cells in the G1 cell cycle phase through regulating cell cycle related proteins like p21. In vivo study confirmed that silencing *CDCA3* could inhibit the proliferation of bladder urothelial carcinoma cells.

**Conclusions:**

*CDCA3* is an important oncogene that could strengthen the migration ability of bladder urothelial carcinoma cells and accelerate tumor cell growth via regulating cell cycle progress and is a potential biomarker of bladder urothelial carcinoma.

**Supplementary Information:**

The online version contains supplementary material available at 10.1186/s12935-021-01969-x.

## Background

Bladder urothelial carcinoma is the most common malignant tumor of the urinary system [1]. Bladder urothelial carcinoma is more common in the elderly, and it could attack both men and women. In the USA, bladder urothelial carcinoma ranks the fifth among the estimated new cancer cases in 2020 [2]. Nearly 81,400 new cases and 17,980 new deaths are estimated in 2020 [2], and the incidence rate increased with age. At present, the diagnose of bladder urothelial carcinoma still depends on transurethral resection and the synergistic use of radical cystectomy with chemotherapy has been the major management option for muscle-invasive bladder urothelial carcinoma patients [3]. Although the application of chemoradiotherapy and targeted drugs improved the prognosis of patients with early bladder urothelial carcinoma, for patients with advanced stage, the recurrence and metastasis rate of the tumor is high, and the prognosis is still poor [4, 5]. For this reason, these features have become hot spots for research, and the identification of practical biomarkers is of great clinical significance [6–9].

CDCA3 (cell division cycle associated protein 3) is also known as Tome-1. Its open reading frame comprises 268 amino acids, which are essential cytoplasmic proteins in cell mitosis. CDCA3 is a kind of FBOX protein, which is essential for cell mitosis. It participates in forming the E3 ligase complex with SKP1 and CUL1 and is related to ubiquitination and degradation of WEE1, a member of the serine/threonine protein kinase family. It also causes dephosphorylation of CDC2 protein in the G2 phase of the cell cycle, which triggers cells to enter mitosis. Studies have shown that the Skp1-Cullin-F-box complex (SCF) imbalance in controlling the G1/S phase may also lead to the occurrence of human tumors [10–14]. Researches have confirmed the uncontrolled elevation of CDCA3 exerted a specific effect on tumor progression. Adams et al. [10] found that CDCA3 protein was expressed in 81.1% of lung adenocarcinoma and 61.9% of lung squamous cell carcinoma. CDCA3 was significantly elevated in non-small cell lung cancer (NSCLC) samples, and the higher expression of *CDCA3* was associated with worse clinical prognosis. Pérez-Pea et al. [13] found five cycle regulating genes associated with worse RFS and OS in Luminal type A breast cancer, including *CDCA3*. CDCA3 was also reported to regulate E2F1 [15] or activate the NF-κB signaling pathway by interacting with TRAF2 [16] in colorectal cancer. However, researches about the function of CDCA3 in promoting bladder urothelial carcinoma development and progression are still absent. Therefore, we conducted a series of experiments to explore the role of CDCA3 in bladder urothelial carcinoma.

In our present research, we comprehensively analyzed the expression status of *CDCA3* in bladder urothelial carcinoma tissues and the relationship between its expression level and key clinical characteristics. Molecular biology experiments revealed that silencing the expression of *CDCA3* could impair the migration ability of tumor cells and inhibit tumor cell growth via arresting cells in the G1 cell cycle phase. Our study demonstrated that CDCA3 is a novel potential biomarker of human bladder urothelial carcinoma.

## Methods

### Raw data and process

The transcription profiling data of 33 human cancers (including liver cancer, breast cancer, colon cancer and so on) were downloaded from the UCSC Xena database (https://xenabrowser.net/datapages)*.* Level 3 transcription profiling data of human bladder urothelial carcinoma and clinical data were acquired from the TCGA GDC database. GSE13507 microarray dataset was acquired from the GEO database [17, 18]. The clinicopathological characteristics of bladder urothelial carcinoma patients from TCGA database and GSE13507 dataset were respectively listed in Tables 1 and 2.

**Table 1:** Clinicopathological characteristics statistics of BLCA patients from TCGA

Clinicopathological characteristics	*CDCA3* expression level		Total	*P* value
	Low	High		
Overall	211	165	376	
Gender				
Female	59 (27.96%)	39 (23.64%)	98	0.3430
Male	152 (72.04%)	126 (76.36%)	278	
Grade				
Low	17 (8.06%)	3 (1.82%)	20	0.0075**
High	194 (91.94%)	162 (98.18%)	356	
Stage				
I + II	58 (27.49%)	60 (36.36%)	118	0.0657
III + IV	153 (72.51%)	105 (63.64%)	258	
T stage				
T0 + T1 + T2	72 (34.12%)	50 (30.30%)	147	0.4324
T3 + T4	139 (65.88%)	115 (69.70%)	229	
M stage				
M0	110 (52.13%)	70 (42.42%)	180	0.0615
M1 + MX	101 (47.87%)	95 (57.58%)	196	
N stage				
N0	128 (60.66%)	96 (58.18%)	224	0.6265
N1 + N2 + NX	83 (39.34%)	69 (41.82%)	152	

**Table 2:** Clinicopathological characteristics statistics of patients from GSE13507

Clinicopathological Features	*CDCA3* expression level		Total	*P* value
	Low	High		
Overall	90	75	165	
Gender				
Female	15 (16.67%)	15 (20.00%)	30	0.5804
Male	75 (83.33%)	60 (80.00%)	135	
Grade				
Low	76 (84.44%)	29 (38.67%)	105	< 0.001***
High	14 (15.56%)	46 (62.33%)	60	
Invasiveness				
Muscle invasive	22 (22.44%)	40 (53.33%)	62	< 0.001***
Non-muscle invasive	68 (77.56%)	35 (46.67%)	103	
M stage				
M0	86 (95.56%)	72 (96.00%)	158	0.8878
M1 + MX	4 (4.44%)	3 (4.00%)	7	
N stage				
N0	86 (95.56%)	63 (84.00%)	149	0.0125*
N1 + N2 + N3 + NX	4 (4.44%)	12 (16.00%)	16	
Recurrence				
Yes	22 (24.44%)	14 (18.67%)	36	0.3709
No	68 (75.56%)	61 (81.33%)	129	
Progression				
Yes	11 (12.22%)	20 (26.67%)	31	0.0180*
No	79 (87.78%)	55 (73.33%)	134	

### Gene set enrichment analysis (GSEA)

Biological function and pathways analysis of CDCA3 was performed via GSEA version 4.0.3 [19]. The annotated gene sets c2.cp.kegg.V7.1.symbols.gmt and c5.all.v7.1.symbols.gmt were chosen as the reference gene sets. Gene sets that showed FDR q-val < 0.05 and NES > 0.65 were considered significant.

### Screening of CDCA3 related genes

Linkomics database (http://www.linkedomics.org/) and UALCAN database (http://ualcan.path.uab.edu/) were investigated to explore potential CDCA3-related genes. Only positively related genes with coefficients larger than 0.6 were preserved. An online Venn intersected analysis (http://bioinformatics.psb.ugent.be/webtools/Venn/) was conducted to screen out genes co-existing in the two lists.

### PPI network construction

Cytoscape software version 3.7.2 was used to construct the PPI network based on the STRING database results.

### GO and KEGG analysis

Genes that co-existed in the Linkomics list and UALCAN list were submitted to the DAVID database (https://david.ncifcrf.gov/) to explore the potential bio-function and pathway of the genes.

### GEPIA database analysis

GEPIA database was used to explore the correlation between *CDCA3* and *BUB1*, *CCNB1*, *CDC20*, *CDC25C*, *CDC45*, and *PTTG1*. The correlation coefficient was tested by Spearman’s test. *CDCA3* was presented on the X-axis, and the corresponding six cell cycle related genes were used for the Y-axis.

### Bladder urothelial carcinoma cell lines

Bladder urothelial carcinoma cell lines T24 and 5637 were purchased from Chinese Academy of Sciences (Shanghai, China), and were maintained in RPMI-1640 medium (Gibco, Shanghai, China), added with 10% fetal bovine serum (FBS) (Gibco, Melbourne, Australia). Both cell lines were cultured in a humidified atmosphere of 5% CO_2_ and 95% air in a 37 °C incubator.

### Total mRNA extraction

Total RNA was extracted from bladder urothelial carcinoma cells using Qiagen RNeasy Mini Kit (Qiagen, Cat. #74101), and QIA shredder from Qiagen (Qiagen, Cat. #79654) was used to extract mRNA in bladder urothelial carcinoma cells. Genomic DNA contamination was removed using DNaseI digestion (Qiagen, Cat. #79254) in each RNA sample. The quantity control of extracted RNA was evaluated by NanoDrop ® ND-2000 UV-Vis spectrophotometer (Thermo Scientific).

### qRT-PCR

1 µg total RNA was used to synthesize cDNA with Revert Aid Aceq PCR RT kit (Toyobo). The final reaction system with iQ™SYBR®-Green Supermix (Bio-Rad) was 20 µL added with 1 µg cDNA. Primer sequences used were presented in Additional file 1: Table S1.

### *CDCA3* target siRNA transfection

The sense sequence of *CDCA3* target siRNA (si-*CDCA3*) was summarized in Additional file 1: Table S2. si-*CDCA3* and si-control were added to the medium when the cell density reaches 30–40%. After siRNA treatment for 48 h, the altered mRNA and protein levels of CDCA3 were tested by Western blot and qRT-PCR analyses.

### Clone formation assay

About 1000 T24 or 5637 cells per well were cultured in 6-well plates and were placed in the incubator for 10 days. 4% PFA was used to fix colonies for 1 h, and crystal violet was used to stain colonies for 30 min.

### Methyl thiazolyl tetrazolium (MTT) assay

3000 bladder urothelial carcinoma cells per well were placed into 96-well plates combined with 200 µL 1640 medium and were incubated for 5 days at 37 °C. After adding 20 µL methyl thiazolyl tetrazolium per well and being placed in the incubator for 4 h, the medium was discarded, and 200 µL DMSO was added to dissolve the precipitate. Absorption value at 490 nm was detected using a microplate reader (Cat. no. SpectraMax M2; Molecular Devices, Berkeley, CA, USA).

### Migration ability assays

5637 bladder urothelial carcinoma cells were suspended with the amount of 60,000–80,000 cells per 200 µL, and T24 bladder urothelial carcinoma cells were suspended with the amount of 40,000–60,000 cells per 200 µL. Bladder urothelial carcinoma cells cultured in the upper room without FBS and 1640 medium with 10% FBS were added to the bottom room. After being incubated for 24 h in the incubator, cells that failed to cross the membrane were erased, and cells that crossed the membrane were fixed and stained. Pictures were taken under an inverted phase-contrast microscope.

When the bladder urothelial carcinoma cells density reaches almost 90%, a scratch was made to create a gap. Next, PBS solution was used to mildly rinse cells to eliminate floating cells, then a medium containing 2% FBS was added to allow cells to move to fill the gap avoiding proliferation. Four pre-marked dots around the scratched line were imaged at 0 and 24 h after scratching. The ratio of cell movement was calculated by the average distance between the two cell-free boundary lines after cell migration.

### Flow cytometry analysis

After 48 h transfection of specific siRNA, 5637 and T24 cells were collected and washed by phosphate buffer saline to remove FBS. Cell precipitation was re-suspended with 1×DNA Staining Solution containing propidium iodide and permeabilization solution (Multi sciences, China) and stored in darkness for 1 h. Cell cycle alteration was detected by flow cytometry analysis machine (Cat. #FC500, Beckman, USA).

### Total protein extraction and Western blot assay

According to the ratio of 50:1:1, RIPA buffer, protease inhibitor, and phosphatase inhibitor (Sigma-Aldrich, USA) were mixed and added to lysis cell precipitation for half an hour. The incubation condition was 0℃. Then the solution was centrifuged at 12,000*g* for 15 min, and insoluble matters were discarded.

Total protein was separated by electrophoresis with 10% SDS-PAGE gel and transferred to PVDF membrane (Millipore, USA). 5% fat-free milk powder was dissolved in TBST solution and then was used to incubate the membrane for 2 h. Next, the membrane was incubated with primary antibodies (Additional file 1: Table S3) for 12 h at 4 °C and then with secondary antibodies (Additional file 1: Table S4) at room temperature for 2 h. Bands were visualized and pictured by Biomax MR films (Kodak, Rochester, NY).

### In vivo experiment

The animal experiment was in accordance with animal welfare and European animal care guidelines, and was approved and supervised by the Center for Animal Experiment of Wuhan University (approval no. 2018152). Male BALB/c nude mice were purchased from WTLH Co., Ltd. (Beijing, China). T24 bladder urothelial carcinoma cells were used to construct the in vivo model infected by lentiviral-CDCA3-shRNA and lentiviral-vehicle-shRNA. Infected T24 cells were diluted with 2 × 10^7^/mL cells in 200 µL PBS and were subcutaneously injected into the bilateral abdomen of 6 mice. The tumor size was measured at the 5th day, 14th day, 21st day, 28th day, and 35th day after injection and calculated as: tumor size = (length×width^2^)/2 (mm^3^). The xenograft tumors were examined for 35 days, and the mice were sacrificed, and the xenograft tumors were removed and weighed.

### Immunohistochemistry (IHC) assay

Generally, the xenograft tissues were immersed in a 0.01 M citrate buffer (pH6.0) for 10 min after hydration and embedding. After three times of gentle rinsing with PBS, the tissue sections were immersed in 3% H_2_O_2_ at 37 ℃ for 15 min and incubated with primary antibody (Proteintech, China, Cat. 15594-1-AP) at 4 ℃ for 12 h. After re-washing, secondary antibodies were co-incubated with tissue sections for 30 min, followed by DAB substrate chromogenic agent and HRP substrate solution for 30 min, hematoxylin was re-stained for 1 min, and final dehydration was carried out. Photos were taken under a microscope, and subsequent analysis was conducted.

### Statistical analyses

All experiments were performed three times, and representative data were selected from the three repeats. Two-tailed Student’s t-test was used to evaluate the statistical significance of differences between control and test groups. Statistical analyses were conducted by SPSS 16.0, and P < 0.05 was regarded as statistically significant.

## Results

### *CDCA3* was significantly elevated in bladder urothelial carcinoma

As several articles have reported that *CDCA3* was a ubiquitous proto-oncogene of human cancers, we investigated the Oncomine database to explore the expression level of *CDCA3*. Results from the online database showed that the expression level of *CDCA3* was elevated in many types of human cancers such as breast cancer, colorectal cancer, and bladder urothelial carcinoma (Additional file 1: Fig. S1 A). We again explored the interactive bodymap in the GEPIA database, where we got a similar result (Additional file 1: Fig. S1B). Moreover, for reconfirmation, we downloaded the expression profiles of 33 human cancers (including liver cancer, breast cancer, colon cancer and so on) from the UCSC Xena database and comprehensively analyzed the mRNA level of *CDCA3* in various human cancers. Our analysis results demonstrated that the expression level of *CDCA3* mRNA was obviously up-regulated in 18 types of human cancer, including bladder urothelial carcinoma (Additional file 1: Fig. S1 C).

**Fig. 1:**
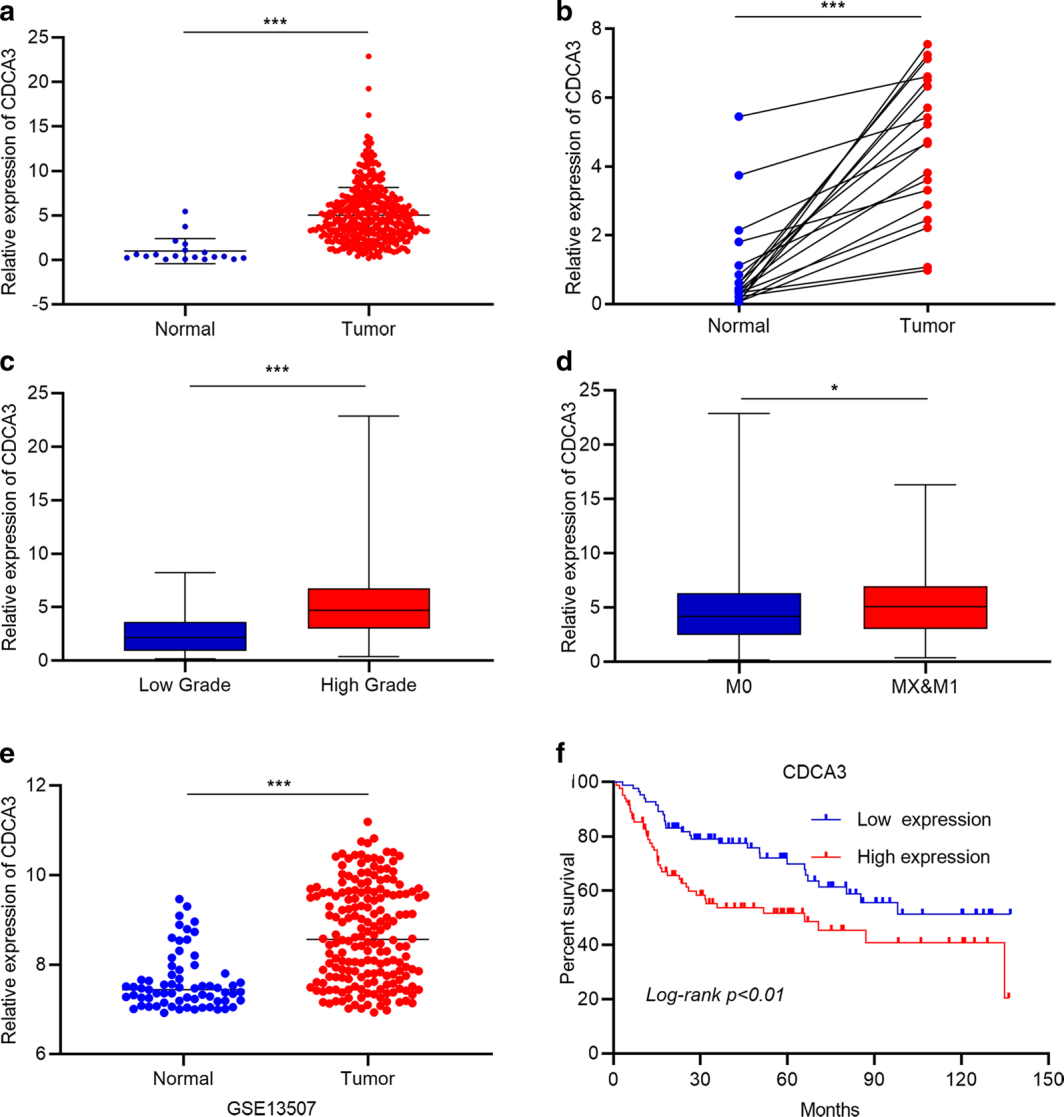
*CDCA3* was related to crucial clinical characteristics and was a potential predictor of bladder urothelial carcinoma. **a** *CDCA3* was significantly elevated in bladder urothelial carcinoma samples collected by the TCGA GDC database. **b** The expression of *CDCA3* was higher in tumor tissues in the matched pairs. **c** Advanced bladder urothelial carcinoma samples showed a higher expression level of *CDCA3*. **d** Samples with distant metastasis presented a higher status of *CDCA3*. **e** *CDCA3* was obviously higher in bladder urothelial carcinoma tissues than in normal bladder tissues, according to the GSE13507 microarray dataset. **f** The survival analysis of *CDCA3* expression status with overall life span in bladder urothelial carcinoma patients. *P < 0.05, ***P < 0.001

Next, we chose to examine the correlation of the level of *CDCA3* with crucial clinical characteristics in bladder urothelial carcinoma, such as tumor grade, tumor distant metastasis, and the overall survival rate of bladder urothelial carcinoma patients. We used data from the TCGA GDC database and GEO database. The results showed that *CDCA3* demonstrated a significantly high expression in the whole samples, and in the matched pairs, the mRNA level of *CDCA3* was also higher in bladder urothelial carcinoma tissues than in normal tissues (Fig. 1 a, b). Moreover, as for the clinical characteristics, we found a higher mRNA level of *CDCA3* in high grade bladder urothelial carcinoma tissues (Fig. 1c), indicating the potential role of *CDCA3* in bladder urothelial carcinoma development. Chi square test for the correlation between *CDCA3* and clinical characteristics of bladder urothelial carcinoma patients from TCGA database showed that the expression level of *CDCA3* was highly related to tumor grade (Table 1). Also, *CDCA3* was up-regulated in tissues with distant metastasis, suggesting that *CDCA3* may participate in the epithelial-to-mesenchymal transition (EMT) process in bladder urothelial carcinoma (Fig. 1d). Furthermore, the GSE13507 dataset was used for confirmation. Data in GSE13507 verified that *CDCA3* was elevated in bladder urothelial carcinoma tissues (Fig. 1e) and demonstrated that patients with shorter survival span tended to present a higher *CDCA3* level (Fig. 1f) which showed that *CDCA3* was also a potential predictor of bladder urothelial carcinoma. Chi square test for GSE13507 dataset further showed that the expression level of *CDCA3* was not only related to clinical grade but also related to muscularis invasion, lymph node metastasis and tumor progression (Table 2).

In conclusion, in this part, we uncovered that *CDCA3* was significantly elevated in human bladder urothelial carcinoma tissues, positively correlated to key clinical characteristics, and could be a predictive biomarker of bladder urothelial carcinoma.

### *CDCA3* mainly functioned by regulating the cell cycle process

Next, we decided to explore how *CDCA3* functions as an oncogene in human bladder urothelial carcinoma. We investigated the LinkedOmics database and got 20,046 genes related to *CDCA3* (Additional file 1: Fig. S2). Among them, 106 genes were positively related to *CDCA3* with a coefficient larger than 0.6. Another 59 genes with a correlation coefficient larger than 0.6 were acquired from the UALCAN database. We then intersected the two gene sets and got 51 co-related genes (Additional file 1: Fig. S3 A), and subsequent KEGG and GO analysis demonstrated that these positively related genes played a crucial role in regulating cell cycle progression and mitosis (Additional file 1: Tables S5 and S6).

**Fig. 2:**
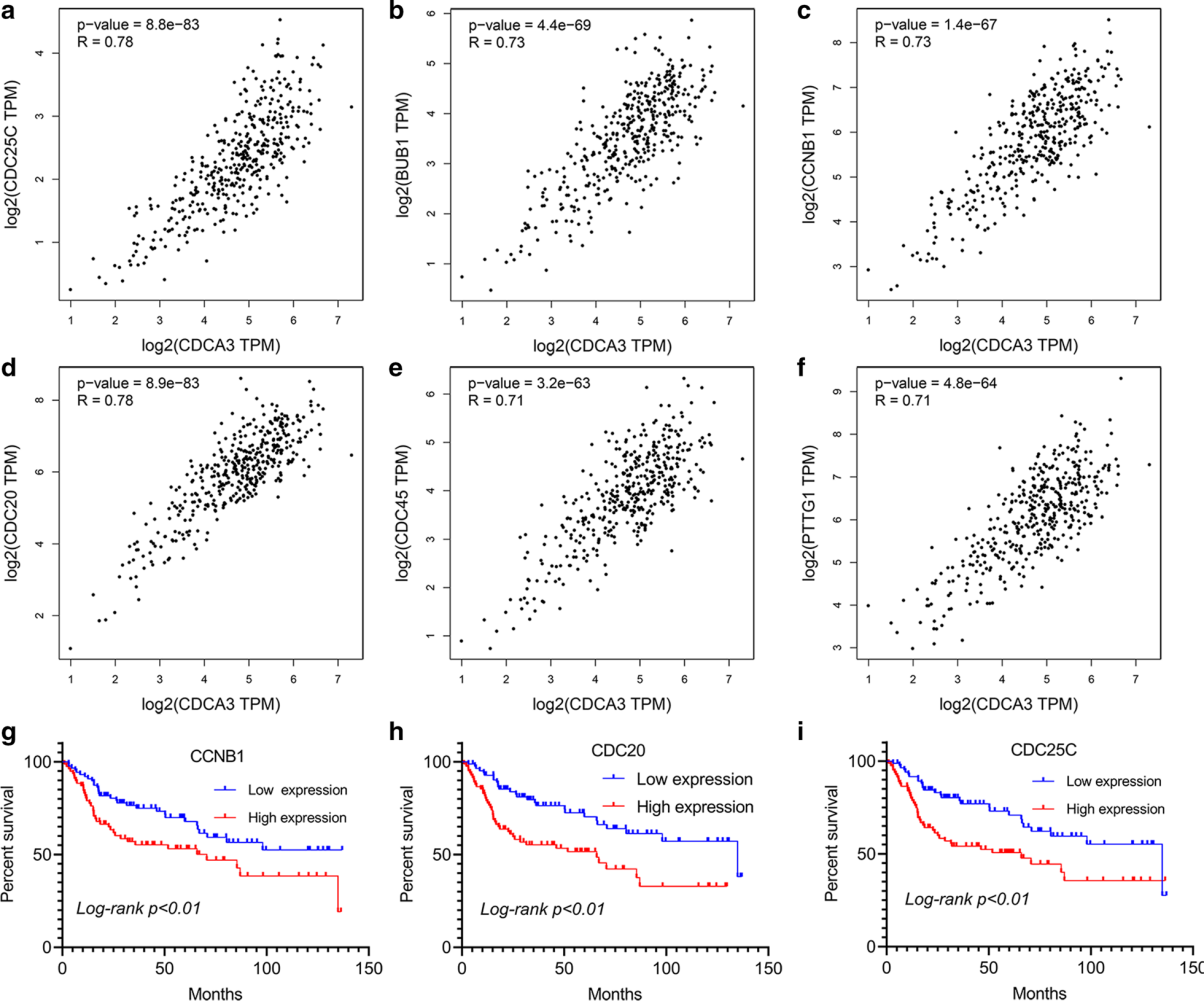
Six cell cycle genes correlated with *CDCA3*. **a–f** Correlation between *CDCA3* and *CDC25C* (R = 0.78), *BUB1* (R = 0.73), *CCNB1* (R = 0.73), *CDC20* (R = 0.78), *CDC45* (R = 0.71) and *PTTG1* (R = 0.71). **g**–**i** Survival analysis of the expression level of *CCNB1*, *CDC20* and *CDC25* in bladder urothelial carcinoma patients

Moreover, to explore the interaction of these genes with *CDCA3*, we constructed a PPI network and found six cell cycle related genes, *BUB1*, *CCNB1*, *CDC25C*, *PTTG1* and *CDC45* had obvious interactions with *CDCA3* (Additional file 1: Fig. S3B). Correlation analysis via the GEPIA database also confirms the intensive relationship between the six genes and *CDCA3* (Fig. 2 a–f). We also explore the predictive value of these six cell cycle related genes and found that *CCNB1*, *CDC20* and *CDC25C* could also predict the overall survival rate of bladder urothelial carcinoma patients (Fig. 2g–i). A GSEA analysis also demonstrated that CDCA3 mainly functioned by regulating the cell cycle process in bladder urothelial carcinoma (Additional file 1: Fig. S4).

Collectively, we demonstrated that *CDCA3* played an oncogenic role through regulating cell cycle and mitosis in human bladder urothelial carcinoma.

### Silencing *CDCA3* could inhibit the proliferation ability of bladder urothelial carcinoma

**Fig. 3:**
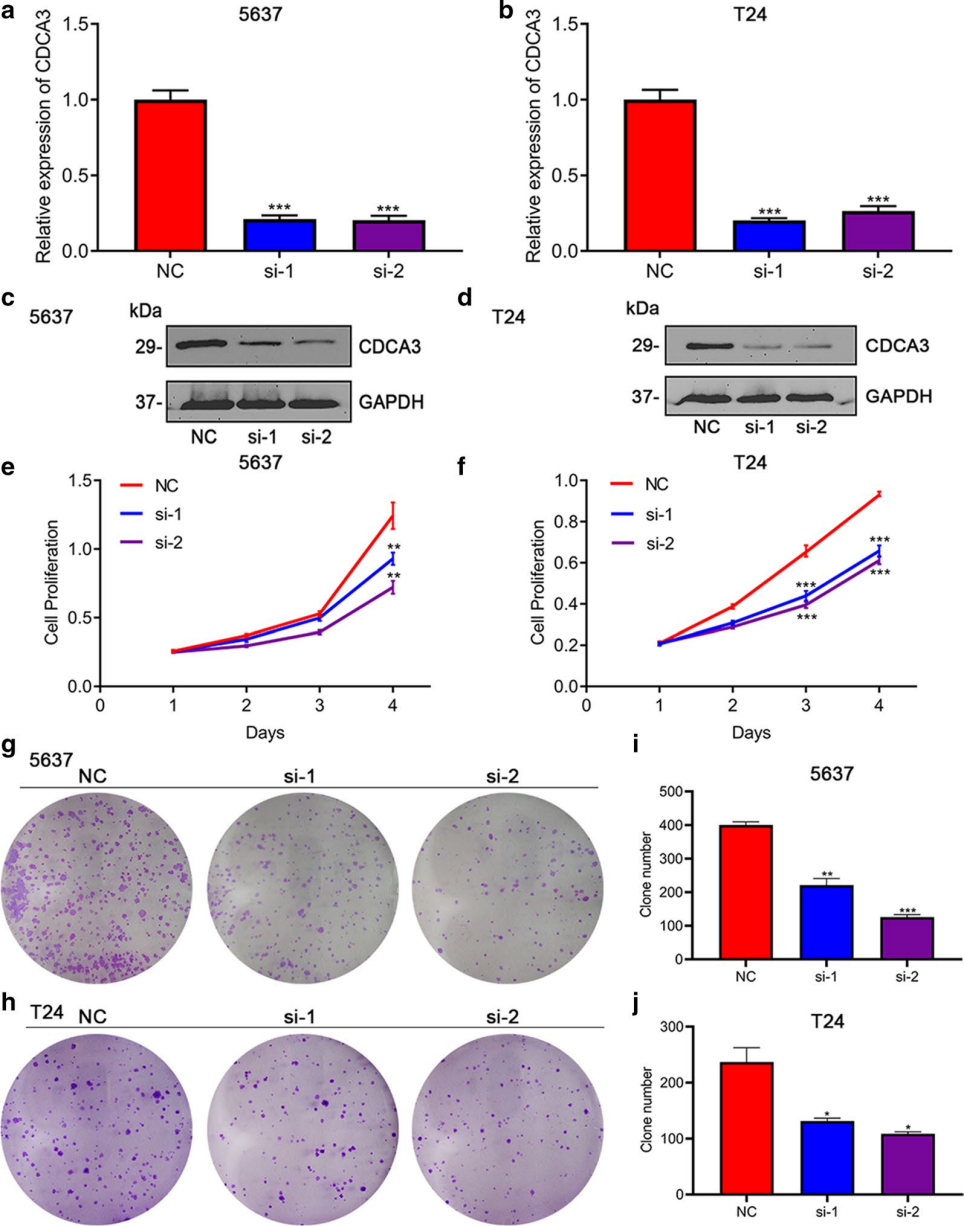
Silencing *CDCA3* significantly inhibited bladder urothelial carcinoma cell proliferation in vitro. The efficiency of two *CDCA3* specific siRNAs in 5637 cell line (**a**) and T24 cell line (**b**) by qRT-PCR. Western blot analysis of the siRNAs treatment in 5637 cell line (**c**) and T24 cell line (**d**). Cell viability tested by MTT assay in 5637 bladder urothelial carcinoma cells (**e**) and T24 bladder urothelial carcinoma cells (**f**). The effect of silencing *CDCA3* on bladder urothelial carcinoma proliferation was tested by clone formation assay in 5637 cells (**g**,** i**) and T24 cells (**h**,** j**). *P < 0.05, **P < 0.01, ***P < 0.001

After the preliminary exploration of the bio-function of *CDCA3*, we tried to further the specific role of the oncogene in promoting bladder urothelial carcinoma. We designed two specific *CDCA3* target siRNAs to transfect 5637 and T24 bladder urothelial carcinoma cell lines. The qRT-PCR (Fig. 3a, b) and Western blot (Fig. 3c, d) analysis showed an obvious decreased expression of *CDCA3* both at the mRNA and protein level after 48 h siRNA transfection compared with the control‐siRNA (NC) group. The cell viability and colony formation assay were conducted to examine whether decreased expression of *CDCA3* could slow down the proliferation rate of bladder urothelial carcinoma cells. Consistent with our expectation, silencing of *CDCA3* could suppress cell growth and colony formation obviously in both cell lines (Fig. 3e–j).

In this part, we constructed two efficient siRNAs to silence the expression of *CDCA3* and subsequent tumor proliferation-related experiments showed that *CDCA3* could promote the growth of bladder urothelial carcinoma.

### Silencing *CDCA3* impaired tumor cell motility via alternating EMT-related proteins

**Fig. 4:**
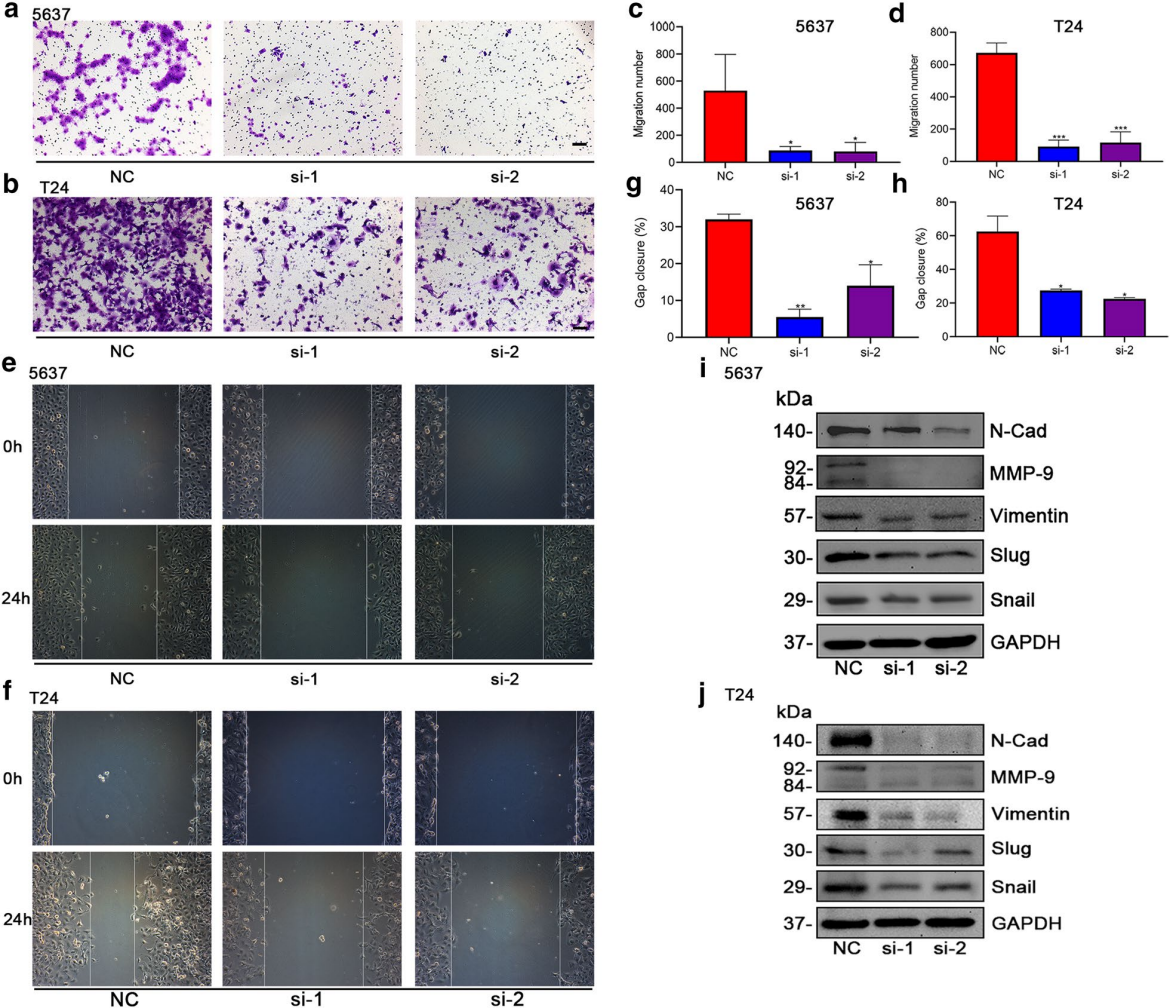
The inhibitory effect of silencing *CDCA3* on cell migration ability. Transwell migration assay performed in 5637 bladder urothelial carcinoma cells (**a**), in T24 bladder urothelial carcinoma cells (**b**) and statistically analyzed (**c**, **d**). The 24-h wound healing assay conducted in 5637 bladder urothelial carcinoma cells (**e**) and T24 bladder urothelial carcinoma cells (**f**) and the statistical analysis (**g**, **h**). Alteration of EMT-related proteins after *CDCA3* specific siRNA transfection in 5637 cells (**i**) and T24 cells (**j**). Scale Bar: 100 μm. *P < 0.05, **P < 0.01, ***P < 0.001

Metastasis, which is closely associated with cell motility, is widely regarded as a malignancy of tumors. EMT is an important event during tumor development and has significance with bad outcomes [20–22]. According to the analysis in Fig. 1d and Table 2, the mRNA level of *CDCA3* may be related to tumor distant metastasis. Thus a transwell assay and wound healing assay were performed to validate the role of *CDCA3* in promoting the migration ability of bladder urothelial carcinoma cells. The transwell assay demonstrated that the migration ability of bladder urothelial carcinoma cells was significantly impaired when *CDCA3* was knocked down (Fig. 4a–d). Likewise, the wound healing assay revealed that the migration rate of 5637 and T24 cells was significantly inhibited after transfection (Fig. 4e, f). The gap closure was calculated in Fig. 4g, h. In addition, key proteins that participate in the EMT progression were analyzed by Western blot assay. Results showed that silencing *CDCA3* induced an obvious reduction of the key EMT markers, such as N-Cad, MMP9, Vimentin, Snail and Slug (Fig. 4i, j).

These two assays revealed that the motility of bladder urothelial carcinoma cells was strongly impaired after the silencing of *CDCA3* and indicated that the potential of CDCA3 in promoting the distant metastasis of bladder urothelial carcinoma.

### Silencing *CDCA3* arrested cell cycle in the G1 phase

**Fig. 5:**
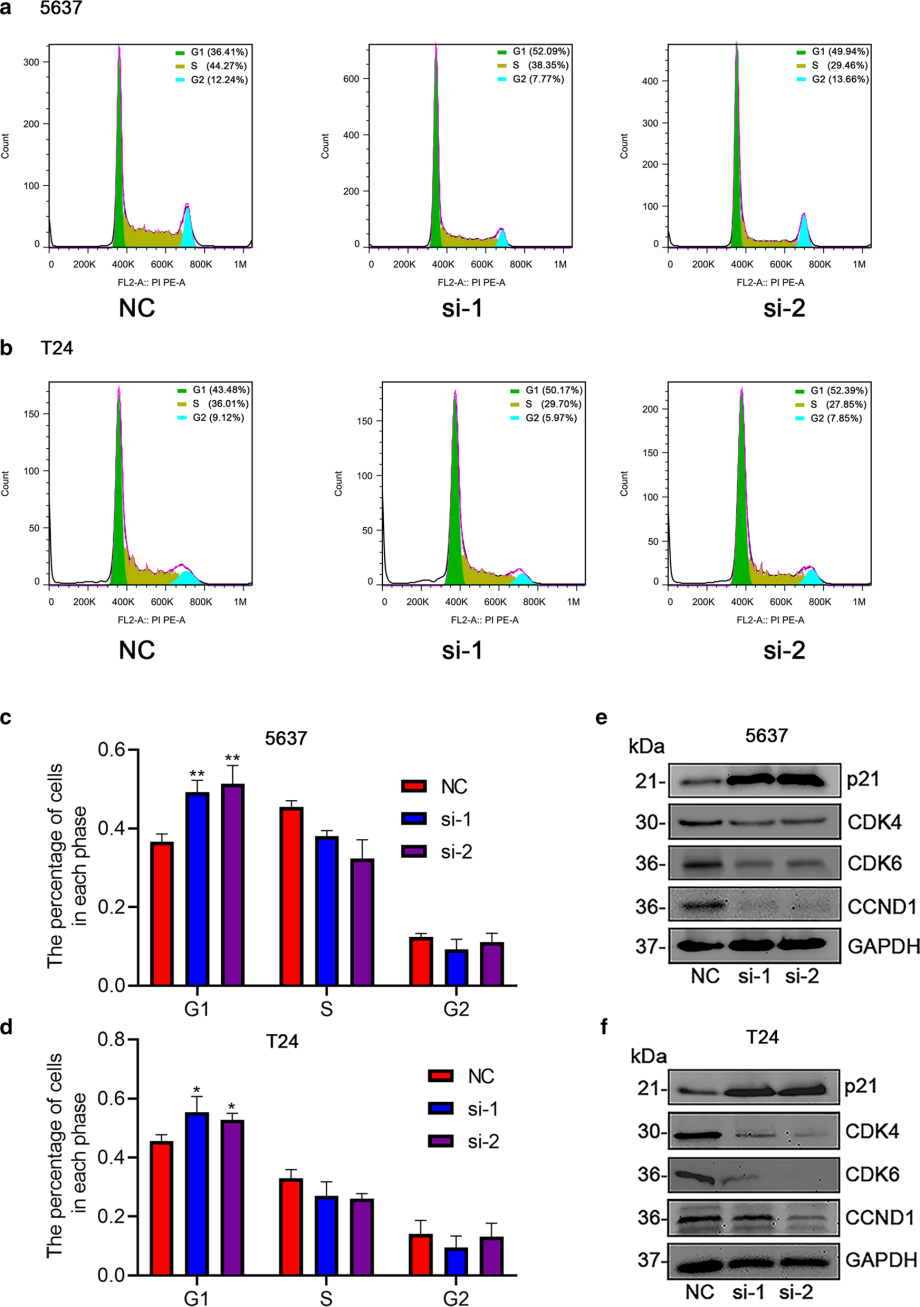
Silencing *CDCA3* arrested bladder urothelial carcinoma cells in G1 phase. Distributions of cell cycles detected by flow cytometry in 5636 cells (**a**) and T24 cells (**b**) after *CDCA3* siRNA treatment and corresponding statistical analysis (**c**,** d**). Alteration of cell cycle related proteins responds to the silence of *CDCA3* in 5637 bladder urothelial carcinoma cells (**e**) and T24 bladder urothelial carcinoma cells (**f**). *P < 0.05, **P < 0.01

As our data have demonstrated that CDCA3 played a significant role in regulating the cell cycle (Additional file 1: Fig. S4), we used flow cytometry to validate this function of CDCA3 in bladder urothelial carcinoma cells. We transfected specific siRNAs to observe whether any changes could be observed. Flow cytometry analysis demonstrated that, as expected, the two types of bladder urothelial carcinoma cells were significantly arrested in G1 phase (Fig. 5a–d). Correspondingly, proteins related to cell cycle such as CDK4, CDK6 and Cyclin D1 were obviously diminished after specific siRNA treatment (Fig. 5e, f), which is consistent with the phenomenon that bladder urothelial carcinoma cells are largely arrested in the G1 phase. Moreover, p21, the well-known inhibitor of CDKs, was strongly up-regulated when *CDCA3* was silenced (Fig. 5e, f), which again confirmed the role of CDCA3 in regulating cell cycle progression.

### Silencing *CDCA3* impaired cell growth in vivo

**Fig. 6:**
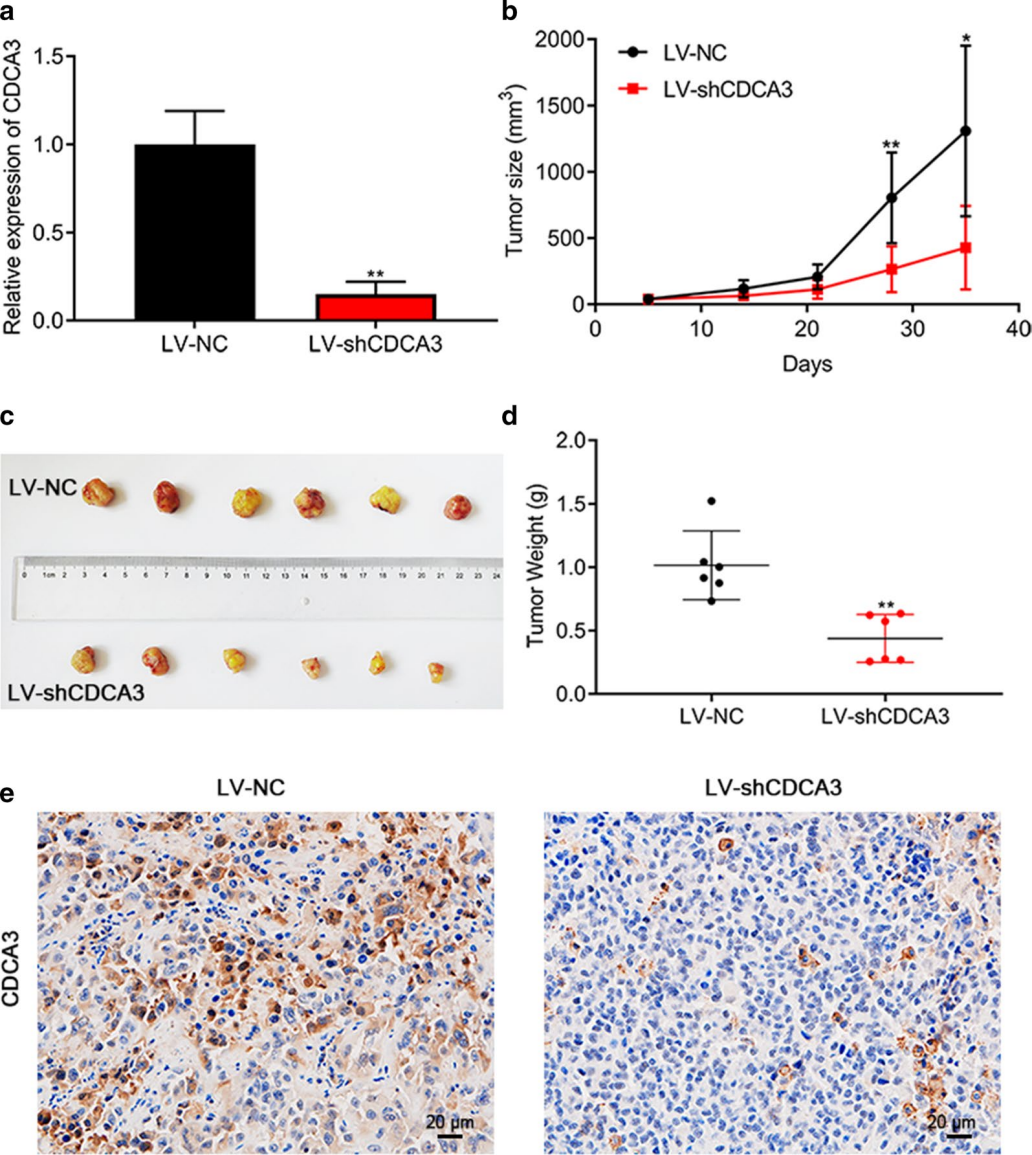
Silencing *CDCA3* significantly inhibited bladder urothelial carcinoma cell proliferation in vivo. **a** The knockdown efficiency of lentiviral-*CDCA3*-shRNA in T24 cells. **b** Xenograft tumor volume was calculated at the 5th day, 14th day, 21st day, 28th day and 35th day after T24 cells were subcutaneously injected. **c** Lentivirus infected T24 cells were subcutaneously injected into BALB/C nude mice. 35 days later, the mice were sacrificed and xenograft tumors were dissected. **d** Weight of the xenograft tumors was measured after mice were sacrificed. **e** Representative IHC images of xenograft tissues from the tumor-bearing mice. Scale bar: 20 μm. *P < 0.05, **P < 0.01

T24 cell line was used to construct an in vivo model via transfecting lentiviral-*CDCA3*-shRNA (Fig. 6a). Five days after the graft was incubated, we observed the grafts were visible and began to measure the graft volume from that time, and the following measurement days were on the 14th, 21st, 28th and 35th day after incubation. Our measurement results showed that on the 28th day the volume of the vehicle treated group was significantly larger than LV-sh*CDCA3* treated group. When the mice were sacrificed the average volume of the vehicle treated group was more than three times that of the LV-sh*CDCA3* treated group, namely 1308 mm^3^ versus 427.3 mm^3^ (Fig. 6b). The results demonstrated that the proliferation speed of the LV-sh*CDCA3* treated group was obviously slowed. Also, when the grafts were stripped, we weighed them and results showed that the average weight of the vehicle treated group was more than twice that of the LV-sh*CDCA3* treated group, namely 1.015 g versus 0.438 g (Fig. 6c, d). Subsequent IHC assay also confirmed that the protein level of CDCA3 was decreased in the LV-sh*CDCA3* treated group (Fig. 6e). In a word, silencing *CDCA3* expression could inhibit the growth ability of bladder urothelial carcinoma cells in vivo.

## Discussion

Based on the analysis of expression profile data of bladder urothelial carcinoma, members of our laboratory have identified several potential biomarkers correlated to the TNM stage and OS of bladder urothelial carcinoma patients, which included CDCA3 [23]. CDCA3, as a member of the FBOX protein family, is the trigger of mitotic entry, which regulates the beginning and end of mitosis [24]. Several previous studies have pointed out that an imbalanced level of *CDCA3* was involved in multiple human tumors [25, 26]. However, due to the heterogeneity in tissue differentiation, the role of CDCA3 in different tumors is diverse. For example, knockdown of *CDCA3* inhibited the growth ability of NCSLC cells via arresting cells in the G2/M phase [10], while in colorectal cancer, CDCA3 could induce cell cycle arrest in the G1 phase [15].

Moreover, besides exerting a cycle-regulating effect, CDCA3 could contribute to chemoresistance in breast cancer [27] and renal cell carcinoma [28]. In addition, the elevated status of *CDCA3* mRNA is regulated by DNA hypomethylation mediated by SP1 [29]. In our present study, we demonstrated that CDCA3 was correlated to bladder urothelial carcinoma progression and could be a novel predictor for bladder urothelial carcinoma patients. A series of functional experiments confirmed that CDCA3 could promote the migration ability of bladder urothelial carcinoma cells and could accelerate cell growth by promoting the course of the cell cycle.

Invasion and distant metastasis are important factors affecting the treatment and prognosis of patients with bladder urothelial carcinoma. Epidemiological studies have shown that approximately 5% of newly diagnosed bladder urothelial carcinoma patients have presented distant metastasis [30]. Although surgical resection and neoadjuvant chemoradiotherapy have achieved certain efficacy, the highly aggressive and metastatic nature of bladder urothelial carcinoma still reduces the efficacy of various treatment methods, leading to dismal outcomes of patients [6, 7, 31, 32]. Metastasis of bladder urothelial carcinoma is a complex and multiple step process. First, the adhesion of bladder urothelial carcinoma cells decreased, and tumor cells began to move out of the original position. Then, tumor cells broke through the basement membrane of the bladder, survived in the lamina propria, and invaded the muscularis propria. With infiltration, the tumor lesion would induce the formation of blood vessels and further promote the metastasis. Bladder urothelial carcinoma cells also could invade blood vessels, survive in blood vessels, travel to distant metastatic organs or tissues, and migrate out from blood vessels to be implanted in organ parenchyma. The factors influencing the above process may exert a specific effect on the metastasis of bladder urothelial carcinoma. As we preliminary demonstrated that CDCA3 might be related to distant metastasis of bladder urothelial carcinoma (Fig. 1d), two migration ability assays were performed to test whether it affected promoting tumor cell migration. Expectedly, the silencing of *CDCA3* significantly decreased the amount of migrated cells and the migration rate of 5636 and T24 cells (Fig. 4a–h). Moreover, the expression level of key EMT-related proteins such as N-Cad, MMP9, Vimentin, Slug and Snail were correspondingly decreased (Fig. 4i, j), which indicated us that the up-regulated CDCA3 could participate in the EMT process of bladder urothelial carcinoma.

The occurrence of the tumor is closely related to abnormal cell cycle regulation. Cyclin dependent kinases (CDKs) are a group of serine/threonine protein kinases. As the engine of cell cycle, CDKs and their regulatory factors exert an important effect in tumorigenesis. CDK4 and CDK6 are important CDKs members that regulated the transition from G1 to S phase. Both kinases are expressed in most cells and bind to Cyclin Ds (D1, D2 and D3) to form CDK4/6-Cyclin complex after being activated by mitotic signals. A growing body of evidence suggested that the over-activated CDK4/6 promoted the development and progression of tumors [33]. In tumor cells, the over-activated CDK4/6 destabilizes the genome and chromosome, leading to uncontrolled proliferation and ultimately abnormal cell cycle regulation. In addition, the activity of CDK4/6 is regulated by Cip and Kip families, especially p21 and p27, and the positive and negative regulation of p21 and p27 can stabilize the CDK4/6-Cyclin complex [34]. According to the KEGG and GO analysis performed online and the GSEA analysis, CDCA3 may function as a cell cycle regulator. To confirm this hypothesis, we silenced *CDCA3* and performed flow cytometry assays to observe the corresponding effect on cell cycle process. As expected, silencing *CDCA3* significantly arrests cell cycle in the G1 phase (Fig. 5a–d) and crucial cycle-related proteins, namely CDK4, CDK6 and Cyclin D1, were decreased consistently (Fig. 5e, f). And p21, as the negative regulator of CDK4/6, was up-regulated after *CDCA3* was silenced. Moreover, as silencing the expression of *CDCA3* could significantly inhibit the proliferation ability of bladder urothelial carcinoma cells both in vitro and in vivo (Figs. 3 and 6), it is reasonable to define that the inhibitory effect of silencing *CDCA3* on cell growth is mainly mediated by arresting bladder urothelial carcinoma cells in G1 phase.

## Conclusions

CDCA3 is an important oncogene that could strengthen the invasive and migrate ability of bladder urothelial carcinoma and accelerate tumor cell growth via promoting the transition from G1 to S phase in bladder urothelial carcinoma. Moreover, as the expression level is closely correlated to the overall life span of bladder urothelial carcinoma patients, CDCA3 exhibited the potential to be a novel biomarker of bladder urothelial carcinoma.

## Supplementary Information

**Additional file 1.** Additional Tables and Figures.

## Data Availability

The data that support the findings of this study are openly available in The Cancer Genome Atlas (TCGA) data portal (https://tcga-data.nci.nih.gov/tcga/) and Gene Expression Omnibus (GEO) database (GSE13507, https://www.ncbi.nlm.nih.gov/geo/query/acc.cgi?acc=GSE13507).

## Abbreviations

CDCA3: Cell division cycle associated protein 3
PPI: Protein–protein interaction
EMT: Epidermal mesenchymal transition
OS: Overall survival.
