# Supplementary material for: The inhibitory effect of silencing CDCA3 on migration and proliferation in bladder urothelial carcinoma
Source: Cancer Cell Int. 2021 May 12;21:257. doi: 10.1186/s12935-021-01969-x (PMC8114508; doi:10.1186/s12935-021-01969-x)
Supplement: Supplementary file 1 — Additional file 1. Additional Tables and Figures. [file 12935_2021_1969_MOESM1_ESM.pdf]

Supplementary data

Supplementary Tables

Supplementary Table 1. Primers for quantitative real-time PCR.

Gene	Forward primer (5′-3′)	Reverse primer (5′-3′)
CDCA3	CTGGAGGGTCTTAAACATGCC	CACTGCTGGTCTTCATAGGTG
GAPDH	TGCACCACCAACTGCTTAG	GATGCAGGGATGATGTTC

Supplementary Table 2. Primers for CDCA3 specific si-RNA.

CDCA3 si-RNA	sequence (5′-3′)
si-1	GCUCUCCUACUCUUGGUAUTT
si-2	GCAAUAGAUGGAAACCAAATT
NC	UUCUCCGAACGUGUCACGUTT

Supplementary Table 3. Primary antibodies.

Antigens	Species antibodies raised in	Dilution (WB)	Supplier
GAPDH, human	Mouse, monoclonal	1:2000	Santa Cruz Biotechnology Inc, USA, cat. no. sc-365062
N-Cad, human	Rabbit, monoclonal	1:1000	Cell Signaling Technology, USA, cat. no. 13116
p21, human	Rabbit, monoclonal	1:1000	Proteintech, China， cat. no. 60214-1-Ig
Slug, human	Rabbit, monoclonal	1:1000	Cell Signaling Technology, USA, cat. no. 9585
CCND1, human	Rabbit, monoclonal	1:1000	Cell Signaling Technology, USA, cat. no. 2978S
CDK4, human	Rabbit, monoclonal	1:1000	Cell Signaling Technology, USA, cat. no. 12790S
CDK6, human	Rabbit, monoclonal	1:1000	Cell Signaling Technology, USA, cat. no. 13331
MMP-9, human	Rabbit, monoclonal	1:1000	Cell Signaling Technology, USA, cat. no. 13667
Vimentin, human	Rabbit, monoclonal	1:1000	Cell Signaling Technology, USA, cat. no. 5741
CDCA3, human	Rabbit, monoclonal	1:1000	Proteintech, China, cat. no. 15594-1-AP
Snail, human	Rabbit, monoclonal	1:1000	Cell Signaling Technology, USA, cat. no. 3879

Supplementary Table 4. Secondary antibodies.

Secondary detection system used	Host	Method	Dilution	Supplier
Anti-mouse IgG (H + L)-HRP	Goat	WB	1:10 000	Sungene Biotech, China, cat. no. LK2003
Anti-rabbit IgG (H + L)-HRP	Goat	WB	1:10 000	Sungene Biotech, China, cat. no. LK2003

Supplementary Table 5. GO function analysis of CDCA3 related genes.

Term	Count	P Value	Genes
GO:0005871~kinesin complex	8	6.47E-11	KIF23, KIF2C, KIFC1, KIF4A, KIF11, KIF18A, KIF18B, KIF20A
GO:0007018~microtubule-based movement	8	1.13E-09	KIF23, KIF2C, KIFC1, KIF4A, KIF11, KIF18A, KIF18B, KIF20A
GO:0005876~spindle microtubule	6	3.27E-08	KIF11, SKA3, NUSAP1, AURKA, SKA1, AURKB
GO:0007076~mitotic chromosome condensation	5	3.42E-08	NCAPH, NCAPG, NUSAP1, CDCA5, NCAPD2
GO:0000070~mitotic sister chromatid segregation	5	3.12E-07	CDCA8, CENPA, NEK2, KIF18A, KIF18B
GO:0007059~chromosome segregation	6	3.13E-07	SPC25, KIF11, HJURP, NEK2, SKA3, SKA1
GO:0000776~kinetochore	6	4.90E-07	CENPO, KIF2C, CENPA, NEK2, KIF18A, AURKB
GO:0005524~ATP binding	15	1.03E-06	KIF23, KIFC1, KIF4A, KIF11, NEK2, KIF18A, KIF18B, AURKA, AURKB, UBE2C, RAD54L, TK1, KIF2C, BUB1, KIF20A
GO:0090307~mitotic spindle assembly	5	1.27E-06	KIFC1, KIF11, NEK2, TPX2, MYBL2
GO:0007080~mitotic metaphase plate congression	5	1.50E-06	KIF2C, KIFC1, CDCA8, KIF18A, CDCA5

Supplementary Table 6. KEGG pathway analysis of CDCA3 related genes.

Term	Count	P Value	Genes
Cell cycle	6	1.32E-06	CCNB1, CDC45, BUB1, CDC20, PTTG1, CDC25C
Oocyte meiosis	5	2.78E-05	BUB1, CDC20, AURKA, PTTG1, CDC25C
Progesterone-mediated oocyte maturation	3	0.00975	CCNB1, BUB1, CDC25C
MicroRNAs in cancer	3	0.02427	KIF23, CDC25C, CDCA5

Supplementary Figures

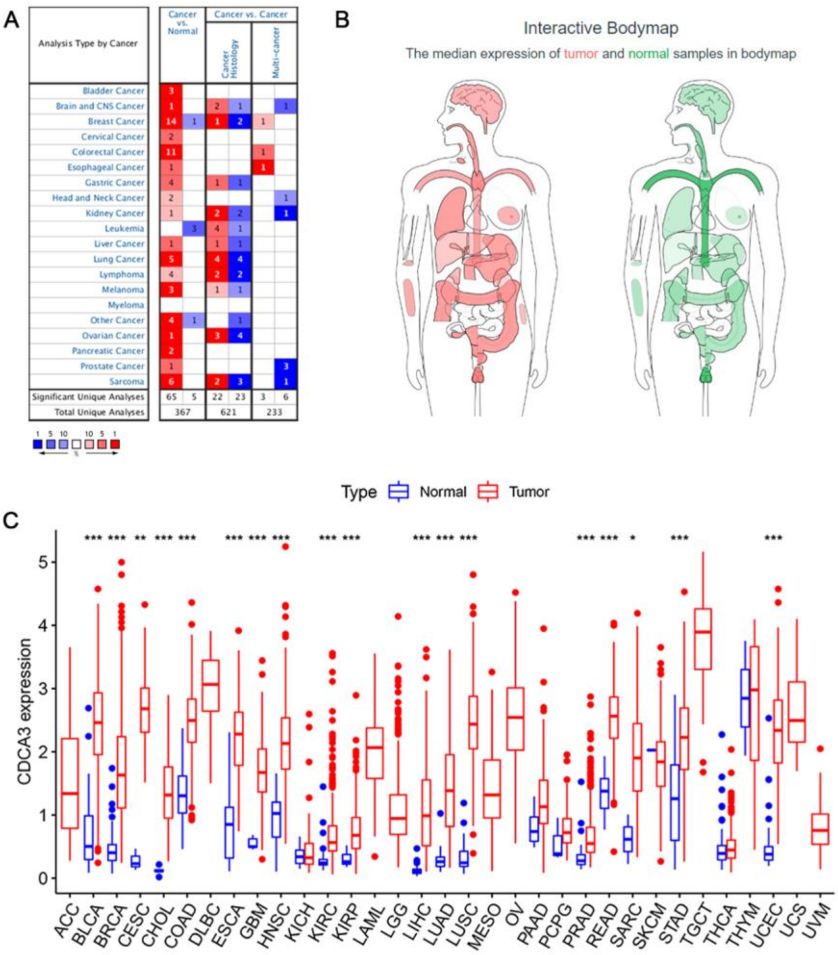

Supplementary Figure 1. The expression status of CDCA3 in human cancers. (**A**) The up- or down-regulated CDCA3 expression in various human cancers compared with corresponding normal samples in the Oncomine database. (**B**) Bodymap of the expression status distributed in the human body. (**C**) The differential expression of CDCA3 in 33 human tumors. *P < 0.05, **P < 0.01, ***P < 0.001.

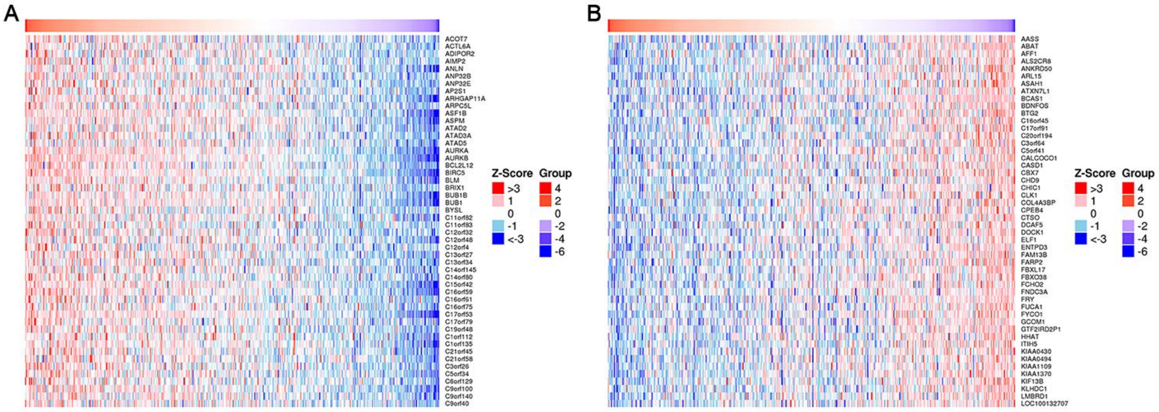

Supplementary Figure 2. Genes were related with CDCA3 via the LinkedOmics database. (**A**) Genes that were positively related to CDCA3. (**B**) Genes that were negatively related to CDCA3.

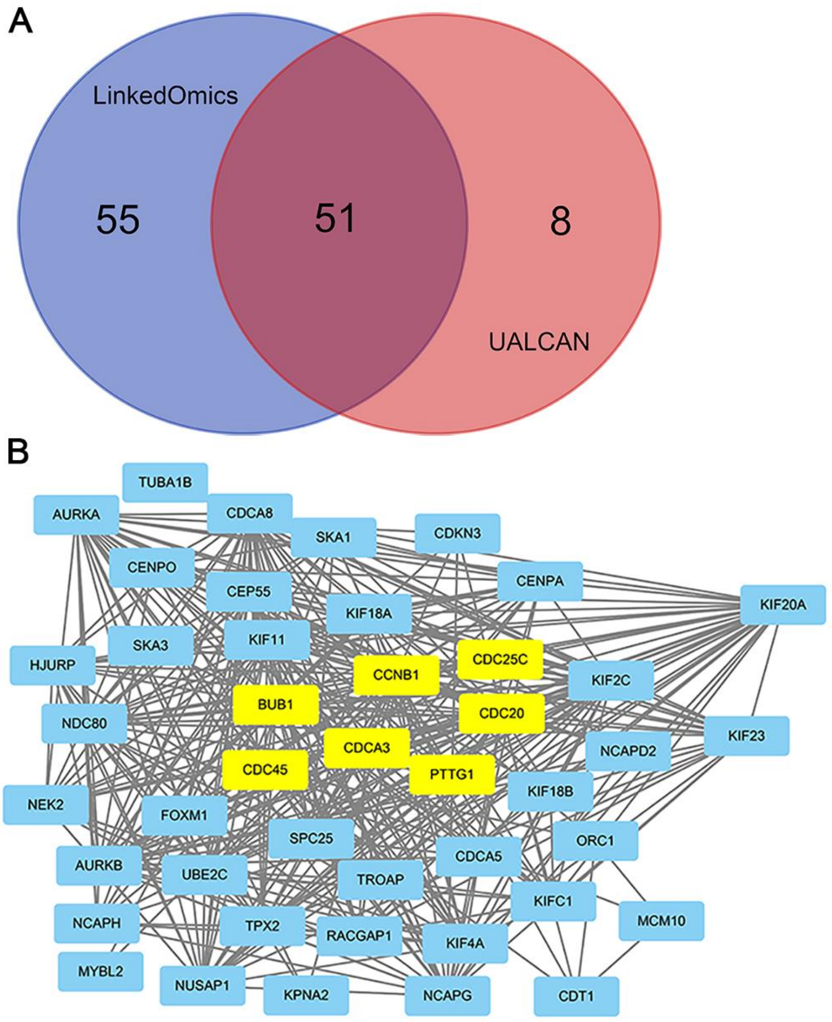

Supplementary Figure 3. The cell-cycle related genes were positively related to CDCA3. (A) The intersected map of LinkedOmics geneset and UALCAN geneset. (B) The PPI network of genes that interacted with CDCA3, and six cell cycle related genes, BUB1, CCNB1, CDC25C, PTTG1 and CDC45 had obvious interactions with CDCA3.

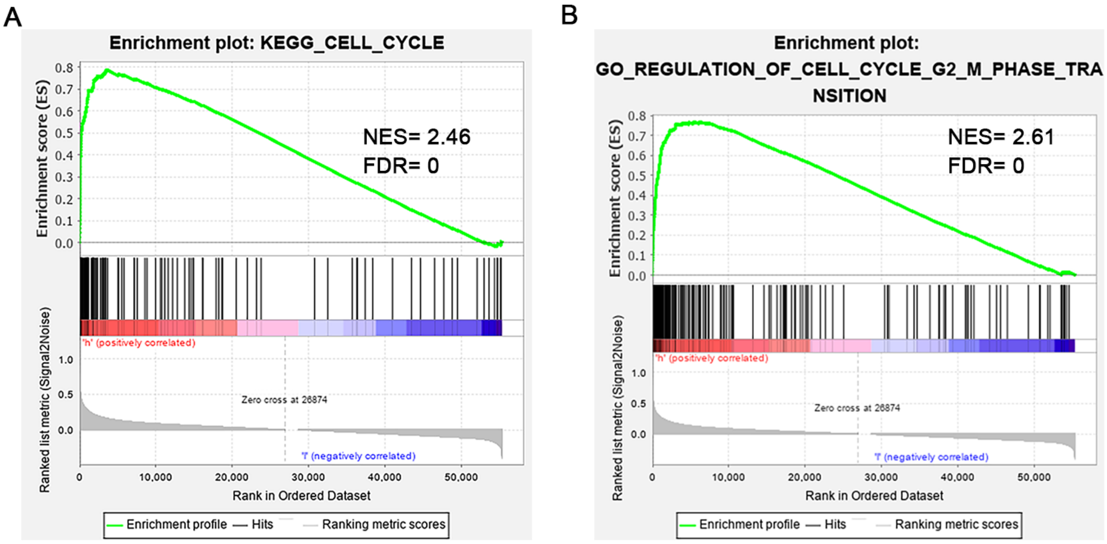

Supplementary Figure 4. CDCA3 mainly functioned by regulating cell cycle process in BCa. (**A**) CDCA3 is significantly related to KEGG CELL CYCLE pathways. (**B**) The GO function analysis of CDCA3.
